# Aerobic Uterine Pathogens in Dairy Cattle: Surveillance and Antimicrobial Resistance Profiles in Postpartum Endometritis

**DOI:** 10.3390/antibiotics14070650

**Published:** 2025-06-26

**Authors:** Ionica Iancu, Sebastian Alexandru Popa, Janos Degi, Alexandru Gligor, Ionela Popa, Vlad Iorgoni, Paula Nistor, Kálmán Imre, Ileana Nichita, Viorel Herman

**Affiliations:** 1 Department of Infectious Diseases and Preventive Medicine, Faculty of Veterinary Medicine, University of Life Science “King Mihai I”, 300645 Timisoara, Romania; ionica.iancu@usvt.ro (I.I.); janosdegi@usvt.ro (J.D.); alexandru.gligor@usvt.ro (A.G.); ionela.popa@usvt.ro (I.P.); vlad.iorgoni@usvt.ro (V.I.); paula.nistor@usvt.ro (P.N.); viorelherman@usvt.ro (V.H.); 2 Department of Animal Production and Veterinary Public Health, Faculty of Veterinary Medicine, University of Life Science “King Mihai I”, 300645 Timisoara, Romania; kalmanimre@usvt.ro; 3 Department of Microbiology, Faculty of Veterinary Medicine, University of Life Science “King Mihai I”, 300645 Timisoara, Romania; ileananichita@usvt.ro

**Keywords:** endometritis, microorganisms, dairy cows, Romania, pathology

## Abstract

Bovine uterine infections remain a widespread challenge in dairy production systems, contributing to reduced fertility and overall herd performance. **Background/Objectives**: Postpartum uterine infections significantly affect dairy cattle fertility and productivity. This study aimed to identify aerobic bacterial pathogens associated with clinical endometritis in Romanian dairy cows and evaluate their antimicrobial resistance profiles. **Methods**: Uterine swab samples (*n* = 348) were collected from clinically affected cows across multiple farms. Bacteria were isolated and identified using conventional culture methods and MALDI-TOF MS. Antimicrobial susceptibility testing was performed using the VITEK^®^ 2 system with GN 96 and GP 79 cards. Statistical analysis was conducted using the chi-square (χ^2^) test. **Results**: A total of 387 bacterial isolates were recovered, with over half of the samples showing mixed bacterial contamination. *Escherichia coli* was the most frequently identified pathogen (44.9%), followed by *Staphylococcus* spp. (17.3%) and *Klebsiella* spp. (14.5%). Gram-negative isolates showed high resistance to tetracycline and ampicillin, while retaining susceptibility to imipenem and polymyxin B. Among Gram-positive isolates, *Streptococcus* spp. were highly susceptible to β-lactams, while *Staphylococcus* spp. showed moderate resistance to penicillin and macrolides. **Conclusions**: This study highlights the prevalence of key aerobic pathogens and their resistance profiles in Romanian dairy herds. These findings support the need for targeted diagnostics and rational antimicrobial use to improve uterine health and therapeutic outcomes in dairy cattle.

## 1. Introduction

Uterine diseases represent a major health challenge in the dairy industry, with a particularly high prevalence among high-producing cows during the postpartum period. These disorders—most commonly metritis and endometritis—not only compromise animal welfare but also contribute substantially to economic losses by impacting the reproductive efficiency and reducing milk production [1,2,3,4]. Metritis is an acute uterine condition that typically occurs within the first 10–14 days postpartum. It is characterized by severe inflammation, an enlarged uterus, fetid discharge, and systemic clinical signs such as fever and reduced milk yield. It is frequently associated with polymicrobial infections, including anaerobic bacteria [5,6]. In contrast, endometritis is a chronic inflammation of the endometrial lining that generally develops after 21 days postpartum. It occurs in the absence of systemic illness and is characterized by persistent purulent or mucopurulent discharge, delayed uterine involution, and reduced fertility. Diagnosis is usually based on vaginal discharge scoring or endometrial cytology. Also, this pathology is defined as an inflammation of the endometrial lining of the uterus, often characterized by degradation of the surface of epithelium, vascular congestion, stromal edema, and infiltration of neutrophils, lymphocytes, and plasma cells [7,8,9]. Clinically, endometritis occurs after calving and leads to delayed uterine involution and late return to estrus, thereby reducing conception rates and increasing the number of inseminations required. Subclinical forms of endometritis are equally negative, as they impact the service period and the viability and quality of the embryo [10].

Epidemiological data indicate that the prevalence of postpartum uterine infections can exceed 50% in herds where calving hygiene, nutritional balance, and reproductive management are suboptimal. These infections are a leading cause of infertility, decreased milk production, and premature culling, resulting in substantial economic losses for the dairy industry [11,12,13].

The uterus is a dynamic microbial environment, particularly during the postpartum period. Microbial contamination is inevitable following parturition, yet not all bacterial presence results in disease and pathologies [14]. The ability of the uterus to restore sterility after calving is a key factor in the development of endometritis. Understanding the complex interactions between host immune responses and the uterine microbiota is essential for identifying cows at higher risk of developing clinical symptoms. Furthermore, elucidating the roles of both commensal and pathogenic microorganisms in the postpartum uterus provides important insights into the mechanisms underlying uterine recovery or the progression to disease [15].

Among the bacteria implicated in uterine infections following calving, aerobic bacteria such as *Escherichia coli* (*E. coli*), *Staphylococcus aureus* (*S. aureus*), *Streptococcus uberis*, and *Proteus* spp. are the most commonly isolated pathogens [16]. These microorganisms isolated from endometritis uterus may act alone or in association, increasing inflammation and tissue damage. For example, *E. coli* acts as a primary colonizer that facilitates secondary infections by other pathogens. Despite widespread antimicrobial use in livestock, treatment outcomes remain inconsistent, largely due to the increasing prevalence of multidrug-resistant bacterial strains, which complicates therapeutic decision-making [17,18]. Similar bacterial species have also been implicated in other diseases in ruminants, such as mastitis in sheep, which shares comparable etiological agents and resistance concerns. Recent studies conducted in Romania have highlighted the presence of *Staphylococcus aureus*, *E. coli*, and *Streptococcus* spp. as major pathogens in ovine mastitis, reinforcing their broader significance in livestock health management [19]. Also, the spread of antimicrobial resistance in veterinary pathogens has implications beyond animal health, with resistant strains potentially entering the food chain or the environment. Similar concerns are seen in human medicine, where antimicrobial-resistant bacteria are a leading cause of nosocomial infections, highlighting the interconnectedness of AMR across sectors [20].

Although advances in veterinary pharmacology have significantly expanded the range of therapeutic options available, clinical outcomes often remain unsatisfactory. A major contributing factor is the growing incidence of antimicrobial resistance among bacterial pathogens associated with endometritis. Untargeted antibiotic use, without prior identification of the causative organisms and their susceptibility profiles, frequently leads to therapeutic failure and fosters the development of multidrug-resistant strains [21]. Consequently, accurate microbiological diagnosis—including the isolation and identification of the pathogens involved—is essential for the formulation of targeted and effective treatment protocols.

Another important aspect is represented by the fact that there is limited information on the specific role of aerobic bacteria in the progression from colonization to clinical disease, particularly in subclinical infections [22]. Additionally, variations in microbial virulence, host immune response, and local environmental factors remain poorly understood. In Romania, research on the bacteriological landscape of bovine endometritis, especially at the regional and farm level, remains insufficient, creating a knowledge gap that obstructs the development of targeted prevention and treatment strategies.

The present study aims to isolate and identify aerobic bacterial species associated with clinical endometritis in dairy cows and to evaluate their potential contribution to disease pathogenesis. By characterizing the bacterial isolates and assessing their antimicrobial susceptibility profiles, the present research provides evidence-based recommendations for effective therapeutic interventions, ultimately leading to improved reproductive performance, reduced economic losses, and more responsible use of antibiotics in dairy herd management.

## 2. Results

A total of 348 uterine swab samples were collected and analyzed from dairy cows of Romanian Spotted (Bălțată Românească), Simmental, and crossbreed lines presenting clinical signs of endometritis, as diagnosed by official veterinarians. So, all uterine swabs were taken from confirmed cases. As such, no negative samples were expected or recorded, and all analyzed specimens resulted in bacterial growth. A total of 387 bacterial strains were isolated, reflecting a significant rate of mixed bacterial contamination and also the complexity of uterine microbial colonization in affected animals. The distribution of bacterial species is presented in Table 1.

The most frequently isolated species was *E. coli*, representing 44.9% (*n* = 174) of all isolates, followed by *Staphylococcus* spp. 17.3% (*n* = 67), *Klebsiella* spp. 14.5% (*n* = 56), *Streptococcus* spp. 13.7% (*n* = 53), and *Pseudomonas* spp. 9.6% (*n* = 37).

Breed-specific distribution of isolates revealed no dominant pattern. Among Romanian Spotted cows, *E. coli* accounted for 14.5% of the total isolates, followed by *Staphylococcus* spp. (7.0%), *Streptococcus* spp. (5.7%), *Klebsiella* spp. (5.4%), and *Pseudomonas* spp. (3.9%). In Simmental cows, the most prevalent isolates were *E. coli* (15.8%) and *Staphylococcus* spp. (4.9%). Crossbreed cows also showed *E. coli* as the leading pathogen (14.6%), followed by *Staphylococcus* spp. (5.4%) and *Klebsiella* spp. (4.7%).

Given the high frequency of mixed bacterial contamination observed in the study, a detailed analysis of the different pathogen combinations recovered from the uterine swabs was performed. Table 2 summarizes the various mono- and polymicrobial infections and their respective prevalence.

Single infections were most frequently associated with *E. coli*, followed by *Streptococcus* spp., *Staphylococcus* spp., and *Klebsiella* spp. Among the mixed infections, the most prevalent combinations included *E. coli* + *Staphylococcus* spp. and *Staphylococcus* spp. + *Klebsiella* spp., indicating common co-infections between Gram-negative and Gram-positive bacteria. While dual-pathogen infections were most frequently observed, several cases involved more complex combinations of three or more bacterial species. In such cases, the total number of isolates exceeded the number of samples due to multiple species being recovered from the same specimen. These findings highlight the microbial diversity present in cases of postpartum endometritis and underscore the importance of considering polymicrobial interactions when evaluating uterine health and designing diagnostic approaches.

Following the identification of bacterial species, antimicrobial susceptibility testing was performed on Gram-negative isolates using the VITEK 2 AST-GN 96 card. The results are summarized in Table 3.

A total of 267 Gram-negative bacterial strains were tested for antimicrobial susceptibility using the VITEK 2 AST-GN 96 card, including *E. coli* (*n* = 174), *Klebsiella* spp. (*n* = 56), and *Pseudomonas* spp. (*n* = 37). Overall, *E. coli* exhibited high susceptibility to imipenem (98.5%), polymyxin B (89.0%), and ceftiofur (85.4%), while showing substantial resistance to ampicillin (65.5%) and tetracycline (76.4%). *Klebsiella* spp. showed complete resistance to ampicillin (100%), which is consistent with intrinsic resistance known for this genus. High susceptibility was observed for polymyxin B (96.4%) and imipenem (100%), while resistance levels for tetracycline and enrofloxacin reached 58.9% and 43.5%, respectively. *Pseudomonas* spp. showed a high resistance profile across most antimicrobials, particularly to neomycin (54.1%), enrofloxacin (54.1%), and flumequine (75.7%). However, these isolates retained relatively high sensitivity to polymyxin B (89.2%) and imipenem (91.9%).

Alongside the investigation on Gram-negative isolates, the present study also assessed the antimicrobial susceptibility of Gram-positive bacterial isolates. These were evaluated using the VITEK 2 AST-GP 79 card, which includes a range of antimicrobials commonly used in veterinary practice. The resistance profiles of *Staphylococcus* spp. and *Streptococcus* spp. are detailed in Table 4.

A total of 120 Gram-positive bacterial strains were tested using the VITEK 2 AST-GP 79 card, comprising *Staphylococcus* spp. (*n* = 67) and *Streptococcus* spp. (*n* = 53). High susceptibility of *Staphylococcus* spp. was identified to gentamicin (85.1%), amoxicillin–clavulanic acid (86.6%), and cefoxitin (79.1%), the latter suggesting a low prevalence of methicillin-resistant strains. Notable resistance was observed to penicillin (64.1%) and tetracycline (49.2%). *Streptococcus* spp. showed even higher susceptibility to penicillin (97.7%), ampicillin (95.3%), and ceftiofur (93.0%), indicating a favorable response to β-lactam antibiotics. However, moderate resistance was noted for tetracycline (53.5%) and erythromycin (30.2%).

## 3. Discussion

To the best of the authors’ knowledge, this is among the few studies conducted in Romania that address the bacterial etiology and antimicrobial susceptibility patterns associated with bovine uterine infections. Considering the growing concern over antimicrobial resistance and its impact on both animal health and treatment efficacy, new and actual data are essential for guiding responsible therapeutic decisions.

Also, the present study provides updated epidemiological insights into the aerobic uterine microbiota associated with postpartum endometritis in dairy cattle from northwestern, western, and southern Romania. The predominant isolation of *E. coli*, consistent with findings from other regions [14,23], highlights its major etiological role in postpartum uterine disease.

The relatively high prevalence of mixed infections, especially those involving *E. coli* and *Staphylococcus* spp., underscores the polymicrobial nature of many uterine infections and the potential for bacterial synergy in exacerbating inflammation and delaying uterine involution [24,25]. Such associations may also complicate antimicrobial treatment strategies, as multidrug resistance patterns may overlap or vary between co-infecting organisms.

The distribution of bacterial isolates across the three cattle breeds (Romanian Spotted, Simmental, and crossbred) revealed no marked dominance by breed, with *E. coli* consistently representing the most frequently isolated pathogen in all groups. This ubiquitous presence of *E. coli* shows its role as a primary etiological agent in bovine endometritis and metritis [26,27]. Although the chi-square test revealed no significant breed-related differences in the distribution of bacterial isolates (χ^2^ = 2.05, *p* = 0.9794), it is important to note that the sample distribution across Romanian Spotted, Simmental, and crossbred cows was not uniform. This reflects the breed structure of the herds involved in the study and may limit the statistical power to detect minor inter-breed variations. Further research using balanced breed representation is warranted to clarify whether genetic or physiological factors influence the uterine microbiota or resistance profiles.

The prevalence of the aforementioned microorganism, ranging from 14.5% to 15.8% across Romanian Spotted, Simmental, and crossbred cows, indicates a widespread environmental exposure and possible host-independent colonization dynamics. These findings are consistent with previous reports identifying *E. coli* as a predominant Gram-negative uterine pathogen, particularly in the postpartum period [26]. Interestingly, *Staphylococcus* spp. and *Klebsiella* spp. were also frequently identified, often in mixed infections. Their distribution showed only minor variation between breeds, suggesting that breed-specific susceptibility is unlikely to be a primary factor in pathogen presence. Also, these bacteria are considered opportunistic pathogens that may contribute to uterine infections under certain conditions, particularly when co-isolated with other uterine microbiota associated with dysbiosis [28].

From a clinical perspective, *Klebsiella* spp. present challenges due to their environmental origin, ability to survive in moist conditions, and intrinsic resistance to multiple antibiotics. The resistance patterns observed in this study emphasize the necessity of targeted antimicrobial susceptibility testing, especially in herds with repeated therapeutic failure. Their involvement in polymicrobial infections could exacerbate inflammatory responses, thereby impairing uterine clearance and delaying recovery of reproductive function [29].

*Streptococcus* spp. and *Pseudomonas* spp., although less prevalent, were also present across all breeds, reinforcing the polymicrobial nature of uterine contamination and the importance of considering both Gram-positive and Gram-negative organisms in therapeutic planning [30]. *Streptococcus uberis* is an environmental pathogen primarily associated with mastitis, but has also been isolated from the uterus of cows with endometritis. Its presence in the uterus is associated with epithelial cell damage and the upregulation of pro-inflammatory cytokines, contributing to the pathogenesis of endometritis. Moreover, *S. uberis* exhibits varying degrees of antimicrobial resistance, particularly to tetracyclines and aminoglycosides, which may complicate treatment protocols [31].

Species of *Staphylococcus*, particularly *S. aureus*, though commonly associated with mastitis, have also been identified in uterine samples of cows with reproductive tract infections, indicating their potential opportunistic role in postpartum endometritis [32,33,34]. The presence of this pathogen in the postpartum uterus may delay uterine involution and has been associated with decreased reproductive performance, even in the absence of clinical signs [33].

The statistical analysis using the chi-square (χ^2^) test revealed no significant association between bacterial species distribution and cattle breed (χ^2^ = 2.05, *p* = 0.9794). This non-significance supports the descriptive observation that bacterial presence is relatively evenly spread across breed groups. The high *p*-value suggests that any observed differences are likely due to chance rather than breed-related biological or immunological factors. It is important to note that while the absence of statistical significance does not negate the biological relevance of microbial presence, it does imply that breed should not be considered an independent predictor of infection type in this context.

The antimicrobial susceptibility profiles of *E. coli*, *Klebsiella* spp., and *Pseudomonas* spp. revealed a concerning prevalence of resistance to multiple commonly used antibiotics. *E. coli* isolates exhibited high levels of resistance to ampicillin (65.5%) and tetracycline (76.4%), consistent with global trends in multidrug resistance among Enterobacteriaceae [35,36]. Resistance to sulfonamides (SXT) and flumequine was also considerable, suggesting reduced efficacy of these agents for therapy. Nevertheless, high susceptibility was maintained for imipenem (98.5%) and polymyxin B (89.0%), which may represent therapeutic alternatives in severe or refractory cases, although their use in veterinary medicine is increasingly scrutinized due to public health concerns [37]. *Klebsiella* spp. showed resistance to ampicillin (100%), as expected, and substantial resistance to fluoroquinolones (e.g., 43.5% for enrofloxacin) and tetracycline (58.9%). Meanwhile, *Pseudomonas* spp. emerged as the most drug-resistant group, showing resistance rates exceeding 50% for multiple antimicrobials, including neomycin, enrofloxacin, and flumequine. The only agents retaining high activity against this genus were imipenem (91.9%) and polymyxin B (89.2%). Statistical comparison of resistance profiles among the three Gram-negative species using the chi-square test demonstrated a highly significant difference (χ^2^ = 121.84, *p* < 0.001). This finding confirms the species-specific nature of antimicrobial resistance and reinforces the need for pathogen-directed treatment. Notably, intrinsic resistance mechanisms in *Pseudomonas* and *Klebsiella*, especially toward β-lactams and tetracyclines, contribute substantially to the observed variance [38].

Among Gram-positive isolates, *Staphylococcus* spp. showed moderate to high resistance to penicillin (64.1%) and tetracycline (49.2%), reflecting known resistance trends among coagulase-positive and coagulase-negative staphylococci. The cefoxitin screening test revealed methicillin resistance in 20.9% of isolates, underscoring the need for routine MRSA surveillance in veterinary pathogens. Encouragingly, susceptibility to ceftiofur (83.6%), gentamicin (79.1%), and florfenicol (71.6%) remained relatively high [39].

*Streptococcus* spp. showed favorable sensitivity to β-lactams (penicillin 90.6%, ampicillin 88.7%), confirming their continued utility in first-line treatments. However, resistance to tetracycline (53.5%) and erythromycin (30.2%) suggests emerging resistance to commonly used broad-spectrum agents. The high susceptibility to ceftiofur (81.1%) and florfenicol (79.2%) aligns with similar findings in recent veterinary studies [40]. A chi-square test comparing antimicrobial resistance between *Staphylococcus* spp. and *Streptococcus* spp. demonstrated a significant difference (χ^2^ = 89.56, *p* < 0.001), particularly in response to β-lactams and macrolides.

Overall, the present study provides a detailed characterization of the bacterial landscape and antimicrobial resistance patterns associated with bovine uterine infections, offering valuable insights into the microbiological dynamics within Romanian dairy herds. While breed-related differences in pathogen distribution were not statistically significant, the presence of multidrug-resistant strains—particularly among Gram-negative isolates—highlights the ongoing challenge of effective antimicrobial management in veterinary practice. These findings serve as a reference point for further research and underline the relevance of evidence-based approaches in reproductive health monitoring.

## 4. Materials and Methods

### 4.1. Study Design

The present cross-sectional study was conducted between 2022 and 2024, with the objective of identifying aerobic uterine pathogens associated with postpartum endometritis in dairy cattle and evaluating their antimicrobial resistance profiles. The study population included dairy cows from 31 farms located in 12 counties from the northwestern, western, and southern parts of Romania. Samples were obtained from dairy farms with herd sizes varying between 50 and 300 cows.

### 4.2. Sample Collection

A total of 348 uterine samples were collected by official vets from dairy cows between 21 and 30 days postpartum presenting clinical signs of endometritis, such as purulent or mucopurulent vaginal discharge and delayed uterine involution. The sampling procedure was performed under aseptic conditions using sterile swabs and sterile gloves. The samples were then transported under refrigeration conditions (0–4 °C) to the Laboratory of Infectious Diseases from the Faculty of Veterinary Medicine, Timisoara. In all cases, swab specimens were delivered to the laboratory within 24 h of collection.

### 4.3. Bioethical Commission Statement

This study was based solely on the analysis of biological material (uterine swabs) collected by official veterinarians as part of routine postpartum clinical examinations conducted for diagnostic purposes. No experimental procedures or direct interventions involving live animals were carried out by the research team. The study design was reviewed and approved by the Bioethics Commission of the University of Life Sciences “King Mihai I” of Timișoara, confirming full compliance with ethical standards for animal research. Ethical approval was granted under document No. 556.

### 4.4. Pathogen Isolation and Identification

Upon arrival at the laboratory, the samples were inoculated onto selective and non-selective media as follows: blood agar (Oxoid Ltd., Thermo Fisher Scientific, Basingstoke, Hampshire, UK), MacConkey agar (Oxoid Ltd., Thermo Fisher Scientific, Basingstoke, Hampshire, UK), CLED (Oxoid Ltd., Thermo Fisher Scientific, Basingstoke, Hampshire, UK), and incubated in aerobic conditions at 37 °C for 48 h. Bacteriological analyses of endometrial samples were performed following standard microbiological procedures. Samples were aseptically inoculated onto blood agar and MacConkey agar plates, which were incubated at 37 °C under both aerobic and anaerobic conditions for 18–24 h to facilitate bacterial growth.

Morphological characteristics of bacterial colonies were initially assessed, followed by Gram staining and hemolysis evaluation on blood agar. For further identification, standard biochemical tests were performed, including catalase, urease, and citrate utilization assays. Targeted identification of common uterine pathogens was carried out, focusing on *E. coli*, *Klebsiella* spp., *Pseudomonas* spp., *Staphylococcus* spp., and *Streptococcus* spp. Identification of *E. coli* and *Klebsiella* spp. was based on lactose fermentation on MacConkey agar, indole production, citrate utilization, and motility tests. *Pseudomonas* spp. was identified by their characteristic pigment production, oxidase positivity, and non-lactose fermenting colonies on MacConkey agar. *Staphylococcus* spp. was differentiated based on Gram-positive cocci morphology, catalase positivity, and mannitol fermentation, while *Streptococcus* spp. was identified through catalase negativity, hemolytic pattern (α or β) on blood agar, and Lancefield grouping when applicable. The total number of bacterial colonies was recorded for each plate.

Pure cultures were obtained through subculturing, and bacterial isolates were subsequently identified using Matrix-assisted Laser Desorption/Ionization–Time-of-Flight Mass Spectrometry (MALDI-TOF MS, Bruker Biotyper, Billerica, MA, USA), following standardized protocols. All isolates were subjected to species-level identification using Matrix-assisted Laser Desorption/Ionization Time-of-Flight Mass Spectrometry (MALDI-TOF MS), employing the Bruker Biotyper system. Identification confidence was interpreted using standard criteria, with scores ≥ 2.000 considered indicative of reliable species-level identification, and scores between 1.700 and 1.999 supporting genus-level resolution.

Quality control was performed using standard reference strains in accordance with CLSI and manufacturer recommendations to ensure the reliability and reproducibility of antimicrobial susceptibility testing. The following quality control strains were employed: *E. coli* ATCC 25922 for Gram-negative Enterobacteriaceae, *Klebsiella pneumoniae* ATCC 700603 for extended-spectrum β-lactamase (ESBL) detection, *Pseudomonas aeruginosa* ATCC 27853 for non-fermenting Gram-negative bacilli, *Staphylococcus aureus* ATCC 29213 for Gram-positive cocci, including methicillin susceptibility testing, and *Streptococcus* pneumoniae ATCC 49619 for β-hemolytic streptococci. All quality control results were within the acceptable ranges specified by CLSI guidelines and VITEK 2 system standards [41].

### 4.5. Antimicrobial Susceptibility Determination

Antimicrobial susceptibility testing (AST) was carried out using the VITEK^®^ 2 Compact system (bioMérieux, Marcy-l’Étoile, France), following the producer’s protocols. Firstly, pure strains were carefully isolated, and bacterial suspensions were prepared in sterile saline solution to achieve a turbidity equivalent to a 0.5 McFarland standard. For the determination of antimicrobial resistance profiles, two different specific cards for Gram-negative (Vitek AST-GN 96) and Gram-positive (Vitek AST-GP 79) bacteria were used. The Vitek AST-GN 96 cards were used for antimicrobial susceptibility of *E. coli*, *Klebsiella* spp., and *Pseudomonas* spp. strains, together with Vitek AST-GP 79 for *Staphylococcus* spp. and *Streptococcus* spp. isolated strains. The used cards for the determination of antimicrobial susceptibility profile included 28 antimicrobials from 9 classes as follows: β-lactams—ampicillin (AMP), amoxicillin/clavulanic acid (AMC), benzylpenicillin (PEN), cefalexin (CN), cefalotin (CF), cefoperazone (CFP), cefquinome (CEQ), ceftiofur (CEF), and ticarcillin/clavulanic acid (TIC); aminoglycosides—amikacin (AMK), gentamicin (GEN), kanamycin (KAN), neomycin (NEO), and streptomycin (STR); macrolides and lincosamides—erythromycin (ERY), clindamycin (CLI), tilmicosin (TIL), and tylosin (TYL); fluoroquinolones—enrofloxacin (ENR), marbofloxacin (MAR), and flumequine (FLU); phenicols—florfenicol (FFC); polymyxins—polymyxin B (PMB); carbapenems—imipenem (IPM); tetracyclines—tetracycline (TET); and sulfonamides—trimethoprim/sulfamethoxazole (SXT).

### 4.6. Statistical Analysis

Statistical analysis was conducted using Pearson’s chi-square (χ^2^) test to compare the distribution of bacterial species in relation to their antimicrobial resistance profiles. For the results, GraphPad software (v 10.1.0) by Dotmatics^®^ (Boston, MA, USA) was used. The two-tailed *p*-value was computed. Differences were regarded as statistically significant at *p* value ≤ 0.05 [42].

## 5. Conclusions

While certain limitations, such as the focus on aerobic bacteria and the absence of molecular resistance profiling, should be noted, they do not diminish the relevance of the present findings. The present study offers one of the few detailed assessments of uterine microbiota and antimicrobial resistance in Romanian dairy cattle, providing updated and locally specific data that can inform both clinical decision-making and future research. By combining pathogen identification with resistance profiling across multiple bacterial species and cattle breeds, this work contributes valuable epidemiological insight into the management of bovine uterine infections. The results highlight the importance of routine bacteriological screening and antimicrobial susceptibility testing as essential tools for guiding targeted and responsible antibiotic use. This evidence-based approach supports reproductive health management and reinforces antimicrobial stewardship efforts at the farm level. Ultimately, the findings lay a strong foundation for ongoing surveillance and offer practical guidance to veterinarians and producers seeking to optimize treatment strategies in line with current resistance trends.

## Figures and Tables

**Table 1:** Distribution of bacterial isolates in uterine samples.

Breed	Bacterial Isolates (*n* = 387)				
	*Escherichia coli* (%)	*Staphylococcus* spp. (%)	*Klebsiella* spp. (%)	*Streptococcus* spp. (%)	*Pseudomonas* spp. (%)
Romanian Spotted	56 (14.5)	27 (7.0)	21 (5.4)	22 (5.7)	15 (3.9)
Simmental	61 (15.8)	19 (4.9)	17 (4.4)	17 (4.4)	12 (3.1)
Crossbreed	57 (14.6)	21 (5.4)	18 (4.7)	14 (3.6)	10 (2.6)
**Total**	**174 (44.9)**	**67 (17.3)**	**56 (14.5)**	**53 (13.7)**	**37 (9.6)**

**Table 2:** Combinations of isolated aerobic pathogens in uterine samples from clinically diagnosed endometritis cases.

Combination Type	Identified Pathogens	No. of Samples	Prevalence (No. of Samples/Total Isolated)	Total Isolated Strains
I	*E. coli*	126	32.5%	**206**
	*Staphylococcus* spp.	20	5.2%	
	*Klebsiella* spp.	15	3.9%	
	*Streptococcus* spp.	26	6.7%	
	*Pseudomonas* spp.	19	4.9%	
II	*E. coli* + *Staphylococcus* spp.	21	5.4%	75 × 2 ^a^ = **150**
	*E. coli* + *Klebsiella* spp.	13	3.4%	
	*E. coli* + *Streptococcus* spp.	7	1.8%	
	*Staphylococcus* spp. + *Klebsiella* spp.	19	4.9%	
	*Streptococcus* spp. + *Pseudomonas* spp.	15	3.8%	
III	*E. coli + Staphylococcus* spp. + *Klebsiella* spp.	4	1.2%	6 × 3 ^b^ = **18**
	*Streptococcus* spp. + *Klebsiella* spp. + *Pseudomonas* spp.	2	0.5%	
IV	*E. coli* + *Staphylococcus* spp. + *Klebsiella* spp. + *Streptococcus* spp.	2	0.5%	2 × 4 ^c^ = **8**
V	*E. coli* + *Staphylococcus* spp. + *Klebsiella* spp. + *Streptococcus* spp. + *Pseudomonas* spp.	1	0.3%	1 × 5 ^d^ = **5**

Legend: In mixed infections, the total number of isolated strains was calculated based on the number of pathogens detected per sample. ^a^—each sample contained 2 different bacterial species, resulting in 2 isolates per sample; ^b^—each sample contained 3 different bacterial species, resulting in 3 isolates per sample; ^c^—each sample contained 4 different bacterial species, resulting in 4 isolates per sample; ^d^—the sample contained 5 different bacterial species, resulting in 5 isolates in total.

**Table 3:** The antimicrobial susceptibility test results for Gram-negative isolated strain.

Antimicrobial		Species (No. Tested Strains)								
		*Escherichia coli* (*n* = 174)			*Klebsiella* spp. (*n* = 56)			*Pseudomonas* spp. (*n* = 37)		
Class	Substance	S (%)	I (%)	R (%)	S (%)	I (%)	R (%)	S (%)	I (%)	R (%)
β-lactams	AMC	143 (82.2)	13 (7.5)	18 (10.3)	45 (80.3)	5 (8.8)	6 (10.9)	2 (5.4)	1 (2.7)	34 (91.9)
	AMP	49 (28.0)	11 (6.5)	114 (65.5)	-	-	56 (100)	-	-	37 (100.0)
	CN	123 (70.7)	13 (7.6)	38 (21.7)	27 (48.2)	11 (19.6)	18 (32.1)	2 (5.4)	2 (5.4)	33 (89.2)
	CFL	119 (68.4)	16 (9.0)	39 (22.6)	30 (53.6)	10 (17.9)	16 (28.6)	3 (8.1)	2 (5.4)	32 (86.5)
	CFP	136 (78.3)	10 (5.7)	28 (16.0)	33 (58.9)	8 (14.3)	15 (26.8)	6 (16.2)	3 (8.1)	28 (75.7)
	CEQ	133 (76.4)	13 (7.6)	28 (16.0)	39 (69.6)	7 (12.5)	10 (17.9)	5 (13.5)	3 (8.1)	29 (78.4)
	CEF	149 (85.4)	4 (2.5)	21 (12.1)	41 (73.2)	5 (8.9)	10 (17.4)	31 (83.8)	2 (5.4)	4 (10.8)
	TIC	145 (83.2)	11 (6.4)	18 (10.4)	36 (64.3)	9 (16.1)	11 (19.6)	32 (86.5)	2 (5.4)	3 (8.1)
aminoglycosides	GEN	135 (77.5)	10 (5.8)	29 (16.7)	42 (75.0)	2 (3.6)	12 (21.4)	26 (70.3)	1 (2.7)	10 (27.0)
	NEO	94 (54.2)	12 (7.1)	67 (38.7)	38 (67.9)	6 (10.7)	12 (21.4)	15 (40.5)	2 (5.4)	20 (54.1)
fluoroquinolones	ENR	87 (49.8)	6 (3.7)	81 (46.5)	31 (55.4)	1 (1.8)	24 (43.5)	15 (40.5)	2 (5.4)	20 (54.1)
	MAR	107 (61.4)	15 (8.4)	53 (30.2)	36 (64.3)	1 (1.8)	19 (33.9)	17 (45.9)	2 (5.4)	18 (48.7)
	FLU	36 (20.5)	10 (5.9)	128 (73.6)	23 (41.1)	3 (5.4)	30 (53.6)	6 (16.2)	3 (8.1)	28 (75.7)
phenicols	FFC	103 (59.1)	17 (9.9)	54 (31.0)	35 (62.5)	4 (7.1)	17 (30.4)	9 (24.3)	3 (8.1)	25 (67.6)
polymyxins	PMB	155 (89.0)	6 (3.5)	13 (7.5)	54 (96.4)	2 (3.6)	-	33 (89.2)	2 (5.4)	2 (5.4)
carbapenems	IPM	171 (98.5)	3 (1.5)	-	56 (100)	-	-	34 (91.9)	1 (2.7)	2 (5.4)
tetracyclines	TET	32 (18.4)	9 (5.2)	133 (76.4)	20 (35.7)	3 (5.4)	33 (58.9)	11 (29.7)	4 (10.8)	22 (59.5)
sulfonamides	SXT	50 (28.6)	11 (6.3)	113 (65.1)	38 (67.9)	5 (8.9)	13 (23.2)	3 (8.1)	2 (5.4)	32 (86.5)

Legend: S—susceptible; I—intermediate; R—resistant; AMC—amoxicillin/clavulanic acid; AMP—ampicillin; CN—cefalexin; CFL—cefalotin; CFP—cefoperazone; CEQ—cefquinome; CEF—ceftiofur; TIC—ticarcillin/clavulanic acid; GEN—gentamicin; NEO—neomycin; ENR—enrofloxacin; MAR—marbofloxacin; FLU—flumequine; FFC—florfenicol; PMB—polymyxin B; IPM—imipenem; TET—tetracycline; SXT—trimethoprim/sulfamethoxazole.

**Table 4:** The antimicrobial susceptibility test results of Gram-positive isolated strains.

Antimicrobial		Species (No. Tested Strains)					
		*Staphylococcus* spp. (*n* = 67)			*Streptococcus* spp. (*n* = 53)		
Class	Substance	S (%)	I (%)	R (%)	S (%)	I (%)	R (%)
β-lactams	AMP	24 (35.8)	-	43 (64.2)	47 (88.7)	3 (5.7)	2 (4.7)
	PEN	17 (25.9)	7 (10.0)	43 (64.1)	48 (90.6)	4 (7.5)	1 (2.3)
	CFL	51 (76.1)	5 (7.5)	11 (16.4)	40 (75.5)	8 (15.1)	5 (9.0)
	CEQ	52 (77.6)	6 (9.0)	9 (13.4)	n.a.	n.a.	n.a.
	CEF	56 (83.6)	4 (6.0)	7 (10.4)	43 (81.1)	6 (11.3)	4 (7.0)
	OXA	55 (82.1)	4 (6.0)	8 (11.9)	n.a.	n.a.	n.a.
aminoglycosides	GEN	53 (79.1)	4 (6.0)	10 (14.9)	41 (77.4)	7 (13.2)	5 (9.3)
	KAN	49 (73.1)	5 (7.5)	13 (19.4)	n.a.	n.a.	n.a.
	NEO	47 (70.1)	6 (9.0)	14 (20.9)	n.a.	n.a.	n.a.
macrolides	ERY	45 (67.2)	5 (7.5)	17 (25.4)	30 (56.6)	7 (13.2)	16 (30.2)
	TIL	52 (77.6)	5 (7.5)	10 (14.9)	n.a.	n.a.	n.a.
	TYL	50 (74.6)	6 (9.0)	11 (16.4)	n.a.	n.a.	n.a.
lincosamides	CLI	47 (70.1)	6 (9.0)	14 (20.9)	39 (73.6)	10 (18.9)	4 (8.0)
fluoroquinolones	ENR	50 (74.6)	7 (10.4)	10 (14.9)	38 (71.7)	12 (22.6)	4 (7.5)
phenicols	FFC	48 (71.6)	6 (9.0)	13 (19.4)	42 (79.2)	7 (13.2)	4 (7.0)
tetracyclines	TET	29 (43.3)	5 (7.5)	33 (49.2)	18 (34.0)	6 (11.3)	28 (53.5)
sulfonamides	SXT	33 (49.2)	6 (9.0)	28 (41.8)	40 (75.5)	9 (17.0)	4 (8.0)
	Cefoxitin (MRSA)	45 (67.2)	8 (11.9)	14 (20.9)	n.a.	n.a.	n.a.

Legend: n.a.—not available; AMP—ampicillin; PEN—benzylpenicillin; CFL—cefalotin; CEQ—cefquinone; CEF—ceftiofur; OXA—oxacillin; GEN—gentamicin; KAN—kanamycin; NEO—neomycin; ERY—erythromycin; TIL—tilmicosin; TYL—tylosin; CLI—clindamycin; ENR—enrofloxacin; FFC—florfenicol; TET—tetracycline; SXT—trimethoprim/sulfamethoxazole.

## Data Availability

Data are contained within the article.
